# Supplementary material for: Large enhancement of response times of a protein conformational switch by computational design
Source: Nat Commun. 2018 Mar 9;9:1013. doi: 10.1038/s41467-018-03228-6 (PMC5844902; doi:10.1038/s41467-018-03228-6)
Supplement: Supplementary file 1 — Supplementary Information [file 41467_2018_3228_MOESM1_ESM.pdf]

Large enhancement of response times of a protein conformational switch by computational design

DeGrave et al.

Supplementary Figures

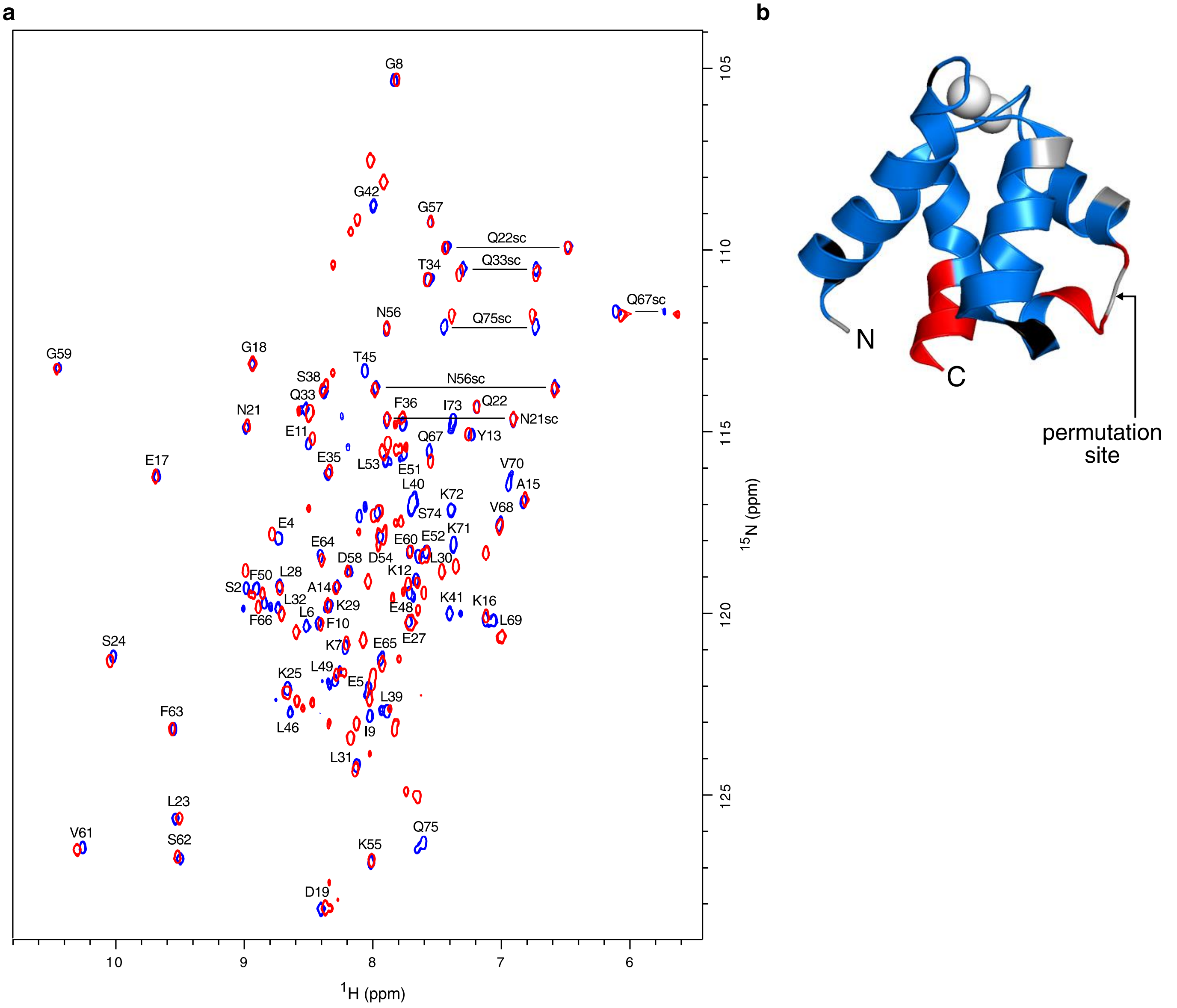

Supplementary Figure 1 | Structural similarity of wild-type and circular permutant calbindin D_{9k} assessed via ^{1}H-^{15}N HSQC NMR. a, Comparison of the ^{15}N heteronuclear single-quantum coherence (HSQC) NMR spectra of WT calbindin (blue) and CP calbindin (red) reveals that most blue cross peaks overlay closely with a corresponding red cross peak, suggesting that the structures of the two proteins are similar at those positions. Blue peaks that do not align with a red peak belong to residues 40-42, 45, and 70-75 of WT calbindin. Blue resonances were cross-assigned by inspection from the HSQC spectrum of WT calbindin.[1] Gln and Asn side chain amide resonances are denoted by "sc". **b,** Residues of WT calbindin whose HSQC cross peaks align with those of CP calbindin are colored blue in the X-ray structure of WT calbindin (PDB 3ICB), and residues 40-42, 45, and 70-75 are shown in red. Structural differences (red) map to areas flanking the permutation site (between residues 43-44) and to the C-terminus of WT calbindin, to which the linker was attached to generate CP calbindin. Gray regions could not be unambiguously

assigned due to peak overlap, and black indicates a Pro residue which lacks an NH cross peak. Calcium ions are shown as spheres.

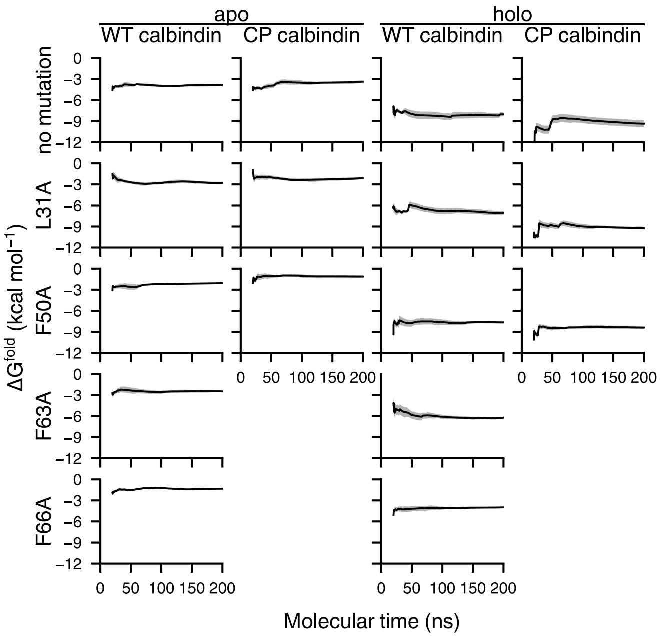

Supplementary Figure 2 | Convergence analysis of folding free energies calculated from weighted ensemble (WE) simulations of the isolated N and N' frames (*i.e.*, WT and CP calbindin). The folding free energy was calculated as $-RT \ln (k_{fold}/k_{unfold})$, where k_{fold} and k_{unfold} are cumulatively averaged starting from 10 ns, and plotted as a function of the molecular time, or Nτ where N is the number of WE iterations and τ is the fixed time interval of each iteration. Error bars (shaded intervals) indicate mean ± s.e.m., calculated via propagation of error; n = 3 independent WE simulations of folding and 3 independent WE simulations of unfolding.

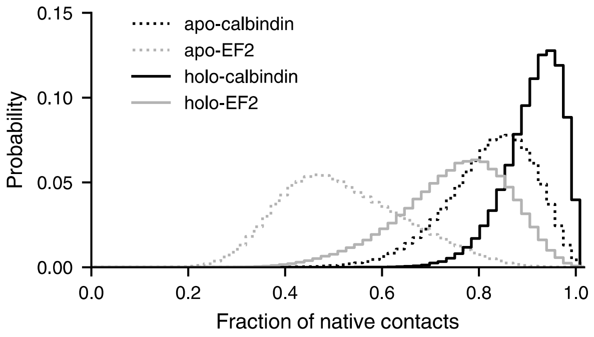

Supplementary Figure 3 | Probability distributions of the fraction of native contacts within the C-terminal EF-hand (EF-2) both as an isolated fragment and in the context of WT calbindin. Data shown is from simulations in the presence (solid lines) and absence (dotted lines) of Ca^{2+}. Probability distributions for apo- and holo-calbindin were each computed from WE simulations and those for apo- and holo-EF2 were each computed from five independent brute-force simulations totaling 25 μs. The probability distributions for apo- and holo-calbindin were generated by first combining 3 independent steady-state WE simulations of both the unfolding and folding processes of the protein to generate an equilibrium set of trajectories and then applying the non-Markovian reweighting procedure to the resulting trajectories to rescale the statistical weights of the trajectories to match their equilibrium values. The reweighting procedure was applied using 1 Å bins along the WE simulation progress coordinate.

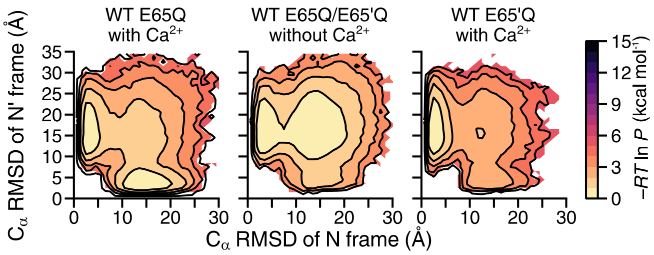

Supplementary Figure 4 | Free energy surfaces for the WT E65Q, E65Q/E65’Q, and E65’Q switch constructs generated at the melting temperature of apo WT calbindin (85 °C).[2] The free energy surfaces reveal that the mechanism of switching differs dramatically from that at the experimental temperature (20 °C). Each free energy surface is based on four, 5-μs standard simulations starting from the folded state N. As done for our simulations at 20 °C, the protein model was parameterized to reproduce the folding free energies of the isolated WT and CP proteins, *i.e.* $\Delta G^{fold} = 0$ at the melting temperature. The resulting parameters were applied to the corresponding frame in the context of the switch construct. The color scale represents free energy and is calculated as $-RT \ln P$, where P is the equilibrium probability density estimated from our simulations.

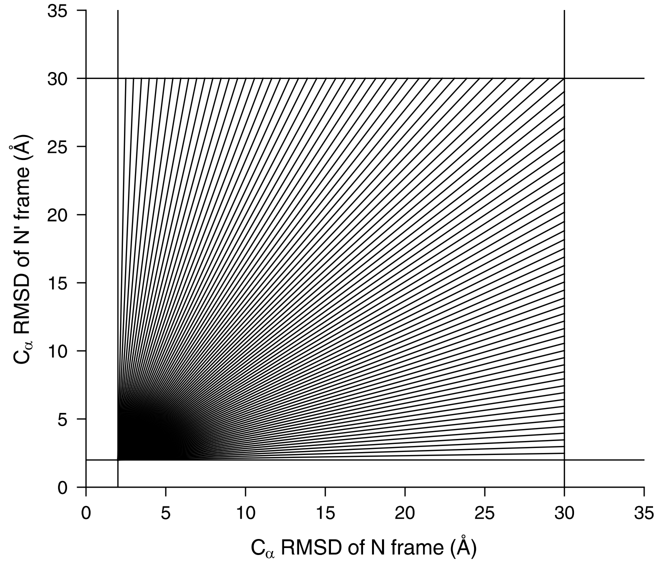

Supplementary Figure 5 | A representative radial binning scheme used for WE simulations of the calbindin-AFF switch constructs. The point from which radial bins emanate was varied to correspond with the probability maxima of the N and N' ground states along the x- and y- axes, respectively.

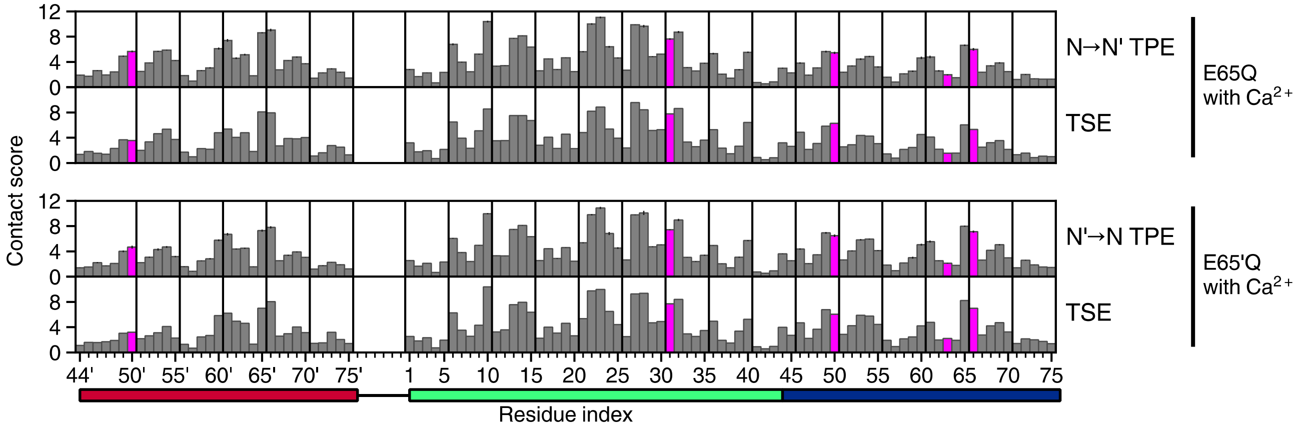

Supplementary Figure 6 | Comparison of contact scores based on the transition path ensemble (TPE) vs. transition state ensemble (TSE). Error bars represent mean ± s.e.m.; n = 10 independent WE simulations for contact scores based on the TPE; n = 146 conformations for TSE of E65Q with Ca^{2+}; n = 107 conformations for TSE of E65'Q with Ca^{2+}. For each switch construct, members of the TSE were identified by first selecting 1000 conformations from the least dense region of the corresponding free energy surface, with the addition criterion that the conformation be part of a successful N → N' or N' → N switching pathway, and then determining the probability of each conformation folding into the target ground state (N or N') before folding into the alternate ground state by running 20 independent standard simulations starting from that conformation. TSE conformations were defined as those having equal probabilities of first folding into the ground state N and first folding into the ground state N' ($0.4 \leq p_N \leq 0.6$, where p_N is the probability that a simulation started from a given conformation will reach N before N').

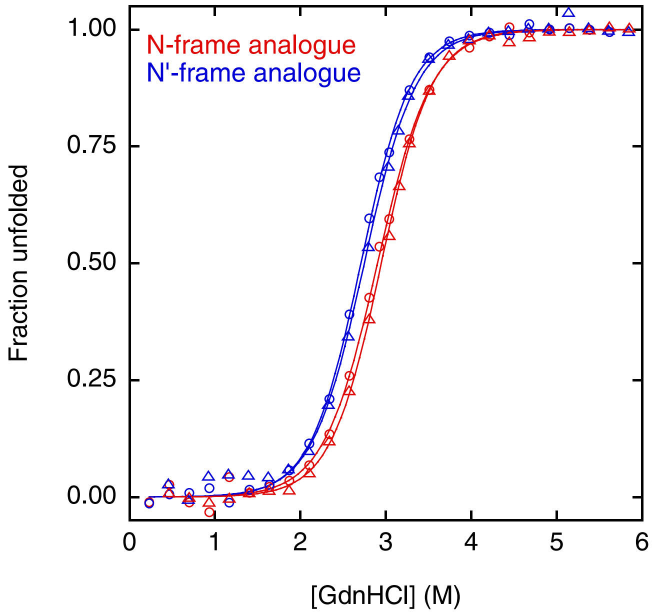

Supplementary Figure 7 | Reversibility of GdnHCl-induced unfolding of N- and N'-frame analogues assessed by circular dichroism experiments. Unfolding (triangles) and refolding (circles) experiments were performed by diluting either native protein (in buffer) or unfolded protein (equilibrated for 3 h in 6.3 M GdnHCl), respectively, into the indicated final concentrations of GdnHCl. The unfolding and refolding curves are coincident, indicating that folding/unfolding are reversible and at equilibrium for both analogues. See main text Methods for experimental details. Solid lines are best fits to the linear extrapolation equation.

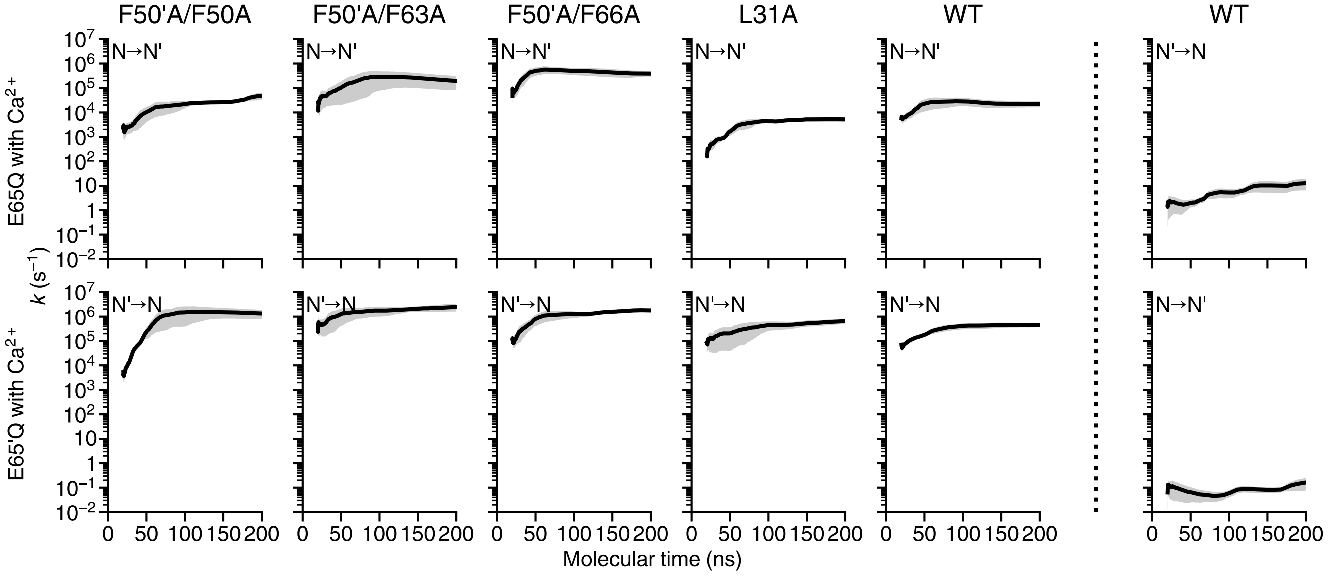

Supplementary Figure 8 | Convergence analysis of switching rate constants calculated from WE simulations. Rate constant estimates are cumulatively averaged from 10 ns and plotted as a function of the molecular time, or Nτ where N is the number of WE iterations and τ is the fixed time interval of each iteration. Error bars indicate mean ± s.e.m.; to enable quick screening of promising mutations, fewer independent WE simulations (n = 3) were performed for mutant constructs than for WT constructs (n = 10).

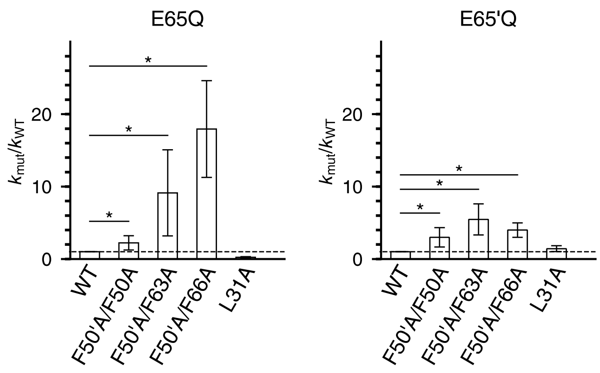

Supplementary Figure 9 | Computed enhancements (k_{mut}/k_{WT}) in switching rate constants of mutant constructs relative to the WT construct (E65Q or E65'Q construct) using molecular simulations. Mutations were modeled by removing all attractive, native interactions involving the residue of interest. All mutations predicted via relative contact scores to improve the response time of the switch (F50'A/F50A, F50'A/F63A, and F50'A/F66A) resulted in $k_{mut}/k_{WT} > 1$ (above the dashed line), while the negative control (L31A) mutation did not significantly increase the switching rate constant. To enable quick screening of promising mutations, fewer independent WE simulations (*n* = 3) were performed for mutant constructs than for WT constructs (*n* = 10). Error bars indicate mean ± s.e.m., calculated by propagation of error. *P < 0.05 in a one-tailed Student's *t*-test.

Supplementary Tables

Supplementary Table 1 | Folding free energies (ΔG^{fold}) of the WT and CP calbindin proteins in the absence and presence of Ca^{2+} (apo and holo forms, respectively), as determined by simulation and equilibrium denaturation experiments. Parameters ε, V_1, and V_3 were chosen to reproduce the experimental folding free energies for apo and holo WT calbindin; experimental values represent WT and CP calbindin without the E65Q mutation. Values indicate mean ± s.e.m.; *n* = 3 independent WE simulations of folding and 3 independent WE simulations of unfolding for values from simulation; *n* = 5 equilibrium denaturation experiments for values from experiment.

		ε (kcal mol^{-1})	V_1 (kcal mol^{-1})	V_3 (kcal mol^{-1})	ΔG^{fold} (kcal mol^{-1}) simulation	ΔG^{fold} (kcal mol^{-1}) experiment (ref. 9)	ΔG^{fold} (kcal mol^{-1}) experiment, this study	m (kcal mol^{-1} M^{-1})	C_m (M)
WT calbindin	apo	0.60	0.50	0.25	-3.9 ± 0.1	-3.92 ± 0.4	-4.95 ± 0.16	1.89 ± 0.09	2.63 ±0.06
	holo	0.71	0.592	0.296	-8.0 ± 0.3	-8.29 ± 0.4			
CP calbindin	apo	0.60	0.50	0.25	-3.4 ± 0.2	-5.54 ± 0.4	-5.86 ± 0.11	2.03 ± 0.04	2.88 ± 0.01
	holo	0.71	0.592	0.296	-9.4 ± 0.5	-7.98 ± 0.4			

Supplementary Table 2 | Rate constants for the N → N' and N' → N switching processes determined by simulation and experiment for Ca^{2+}-bound WT (E65Q, E65'Q) and mutant constructs. Values indicate mean ± s.e.m.; for values from simulation, *n* = 10 independent WE simulations for WT constructs, *n* = 3 independent WE simulations for mutant constructs. For values from experiment, *n* = 15 stopped-flow experiments for all WT E65Q variants and E65'Q/F50'A/F50A; *n* = 17 for WT E65'Q and E65'Q/F50'A/F66A, *n* = 18 for E65'Q/F50'A/F63A, *n* = 12 for E65Q/L31A, and *n* = 11 for E65'Q/L31A. The rate constant for each mutant construct (except E65Q/L31A in experiment, and both L31A constructs in simulation) is significantly greater than that of the corresponding WT construct ($P < 0.05$ in a one-tailed Student's *t*-test).

		Simulation $k_{N\rightarrow N'}$ (s^{-1})	Simulation $k_{N'\rightarrow N}$ (s^{-1})	Experiment $k_{N\rightarrow N'}$ (s^{-1})	Experiment $k_{N'\rightarrow N}$ (s^{-1})
E65Q	WT	(2.2 ± 0.6)×10^4	(1.3 ± 0.6)×10^1	11.7 ± 0.3	
	F50'A/F50A	(4.8 ± 1.6)×10^4		36.6 ± 0.5	
	F50'A/F63A	(2.0 ± 1.2)×10^5		72.3 ± 8.9	
	F50'A/F66A	(3.9 ± 0.9)×10^5		141.2 ± 2.9	
	L31A	(5.2 ± 1.1)×10^3		12.0 ± 0.3	
E65'Q	WT	(1.6 ± 0.9)×10^{-1}	(4.6 ± 0.9)×10^5		1.70 ± 0.02
	F50'A/F50A		(1.3 ± 0.5)×10^6		53.7 ± 0.5
	F50'A/F63A		(2.4 ± 0.8)×10^6		11.3 ± 0.6
	F50'A/F66A		(1.8 ± 0.3)×10^5		35.3 ± 0.5
	L31A		(6.6 ± 1.4)×10^5		2.7 ± 0.3

Supplementary Table 3 | Primer sequences used in this study. CP44, circular permutant calbindin D_{9k}; C44, WT calbindin D_{9k} with Cys inserted at position 44.

Primer name		Sequences
CP44 N-terminal Cys		GATCCATATGTGCAGCACTCTGGATGAACTG
CP44 C-terminus		GATTTCCTCGAGTTACATACCTTTCAGCAGAGACGGG
N-terminus linker		GAAATCGCGGCCGCTATGAAATCTCCGGAAGAACTG
C-terminus linker		GATTTCAGCGGCCGCGCCACCCTGAGAGATCTTCTTAACCAGAACC
L31A	sense	AAACTGGCGCTGCAGACTGAGTTCCCGTC
	anti-sense	CTGCAGCGCCAGTTTCAGTTCTTCTTTAGACAGCTGG
C44	sense	GGTATGTGCAGCACTCTGGATGAACTGTTCG
	anti-sense	AGTGCTGCACATACCTTTCAGCAGAGACGGG
C44 F50A	sense	GAACTGGCCGAAGAACTCGATAAAAACGGTGATGG
	anti-sense	TTCTTCGGCCAGTTCATCCAGAGTGCTGCACATACC
E65Q	sense	GAAGTTTCTTTCGAACAGTTCCAGGTTCTGG
	anti-sense	CCAGAACCTGGAACTGTTCGAAAGAAACTTC
F63A	sense	GTTTCTGCGGAAGAGTTCCAGGTTCTGGTTAAG
	anti-sense	CTCTTCCGCAGAAACTTCACCATCACCGTTTTTATCG
F66A	sense	GAAGAGGCGCAGGTTCTGGTTAAGAAGATCTCTC
	anti-sense	AACCTGCGCCTCTTCGAAAGAAACTTCACC
F63A E65Q	sense	GTTTCTGCGGAACAGTTCCAGGTTCTGGTTAAG
	anti-sense	CTGTTCCGCAGAAACTTCACCATCACCGTTTTTATCG
E65Q F66A	sense	GAACAGGCGCAGGTTCTGGTTAAGAAGATCTCTC
	anti-sense	AACCTGCGCCTGTTCGAAAGAAACTTCACC

Supplementary Notes

Supplementary Note 1 To test the cooperativity of the protein model, we simulated the isolated C-terminal EF hand (EF-2) using the optimal ε value for WT calbindin (see "Parameterization of the model" in Supplementary Methods). Based on CD spectroscopy, the isolated EF-2 hand is unfolded in the absence of Ca^{2+} and folded in the presence of Ca^{2+}, while the same fragment in the context of WT calbindin is folded whether or not Ca^{2+} is present.[3] The extent of structure in EF-2 observed in our simulations is consistent with these experiments (Supplementary Fig. 3) thereby demonstrating that our protein model has captured the highly cooperative nature of the folding process of calbindin. As a further test of the cooperativity, we applied the ε parameters of WT calbindin in the apo and holo forms (Supplementary Table 1) to simulations of its CP in the apo and holo forms, respectively. Consistent with experiments,[4] the stabilities of WT and CP calbindin in our simulations are similar in their apo forms as well as holo forms (Supplementary Table 1 and Supplementary Fig. 2). We also applied the same set of parameters to the E65Q mutants of calbindin and its CP since the E65Q mutation primarily disrupts Ca^{2+} binding and has minimal effects on the stabilities of these proteins ($\Delta\Delta G^{fold}$ = -0.4 and -0.3 kcal mol^{-1} for WT calbindin and its CP, respectively, in both their apo and holo forms).[4]

Supplementary Note 2 As demonstrated by others for protein folding processes,[5] one advantage of using the TPE instead of the transition state ensemble to quantify the kinetic importance of particular residues (*e.g.*, via Φ-values) is that the TPE yields essentially the same predictions as those obtained with the transition state ensemble while avoiding the need to identify transition states. Consistent with these observations, contact scores that were calculated from an ensemble of >100 transition-state conformations matched well with contact scores calculated from the N→N' TPE and the N'→N TPE (Supplementary Fig. 6); transition-state conformations were defined as having an equal probability of next visiting N as N'. As an additional advantage, the TPE not only accounts for single-barrier processes, but step-wise processes with one or more transient intermediates by simultaneously quantifying the contribution of a given residue to the stability of conformations in each of these higher-energy, non-native states. Furthermore, given the diversity of switching pathways from our simulations, it is important to examine the ensemble of pathways rather than a single pathway (*e.g.*, the minimum free energy pathway) when identifying residues that could be mutated to improve the kinetics of the switch.

Supplementary Methods

The protein model and energy function Due to the extremely long-timescales of protein conformational switching processes, our simulations employ minimal residue-level models to enable predictions of promising mutations for improving switch response times within the usual timeframe of experimental tests (approximately two weeks). In these models, each residue is represented by a single pseudo-atom at the position of its C_α atom. For each switch construct, initial models for simulations consisted of one of the two alternate folding frames (N or N') in a folded conformation and the other in an extended backbone conformation; coordinates for these models were generated using the Modeller software package[6] based on coordinates of the folded and extended conformations of the isolated WT and CP forms of calbindin. Coordinates for the folded conformations of both the WT and CP forms were taken from the crystal structure of WT calbindin D_{9k} (PDB code 3ICB[7]). In the folded conformation of CP calbindin, the six-residue loop (GGAAAM) that connects the original N- and C-termini of WT calbindin was modelled using the loopy program of the Jackal software package.[8]

In the present study, a Gō-type potential energy function[9,10] was used to govern the conformational dynamics of the protein model although any energy function can be used with our design strategy. Despite the limitations of Gō-type models, *i.e.* artificially accelerated dynamics due to the neglect of stabilizing non-native interactions,[11,12] such models are useful from the perspective of protein engineering in terms of (i) their abilities to reproduce experimental stabilities of individual switch components by optimizing the primary adjustable parameter (the well-depth ε), and (ii) their abilities to capture the cooperativity of protein folding, yielding fragment stabilities that are consistent with experimental data.[13]

Bonded interactions in the energy function are modeled by standard molecular mechanics terms:

$$E_{\text{bonded}} = \sum_{\text{bonds}} k_{\text{bond}}(r - r_{\text{eq}})^2 + \sum_{\text{angles}} k_{\text{angle}}(\theta - \theta_{\text{eq}})^2 + \sum_{\text{dihedrals}} V_1[1+\cos(\varphi - \varphi_1)] + V_3[1+\cos(3\varphi - \varphi_3)] \quad (1)$$

where r, θ, φ are pseudo-bond lengths, pseudo-angles, and pseudo-dihedrals, respectively; V_1 and V_3 are potential barriers for the dihedral terms. Equilibrium bond lengths (r_{eq}), angles (θ_{eq}), and dihedral phase angles (φ_1 and φ_3) were taken from the crystal structure. The force constants, k_{bond} and k_{angle}, were set to 100 kcal mol^{-1} $Å^{-1}$ and 20 kcal mol^{-1} rad^{-1}, respectively. To allow free rotation about bonds in the linker region, which was assumed to be flexible, V_1 and V_3 were set to zero for dihedrals involving linker residues.

Nonbonded interactions between residues separated by four or more pseudo-bonds were treated as either native or non-native contacts. A native contact was defined as a residue-residue contact in which the heavy atoms of the two residues are within 5.5 Å of each other in the crystal structure of WT calbindin. Native contacts were modeled using a Lennard-Jones-like potential:

$$E_{ij}^{\text{native}} = \varepsilon^{\text{native}} \left[5\left(\frac{\sigma_{ij}^{\text{native}}}{r_{ij}}\right)^{12} - 6\left(\frac{\sigma_{ij}^{\text{native}}}{r_{ij}}\right)^{10} \right] \quad (2)$$

where $\varepsilon^{\text{native}}$ is the energy well depth for the native contact, r represents interatomic distance during simulation, and σ^{native} represents the corresponding distance in the crystal structure. Non-native contacts were modeled using a purely repulsive potential:

$$E_{ij}^{\text{non-native}} = \varepsilon^{\text{non-native}} \left(\frac{\sigma_{ij}^{\text{non-native}}}{r_{ij}} \right)^{12} \quad (3)$$

where $\sigma_{ij}^{\text{non-native}}$ and $\varepsilon^{\text{non-native}}$ were set to 4.0 Å and 0.10 kcal mol^{-1}, respectively. The WT, CP, and AFF versions of calbindin consisted of 213, 217, and 333 native contacts, respectively. To prevent formation of unphysical conformations involving "mirror image" contacts between EF-2 and EF-2', native contacts between EF-2 and EF-2' were excluded from the model.

The energy well depth $\varepsilon^{\text{native}}$ was tuned to reproduce the stabilities of apo and holo WT calbindin, as described below in "Parameterization of the protein model", applying appropriate parameters to the Ca^{2+}-bound frame and unliganded frame of each AFF construct. For native contacts of residues in the shared region of the switch (EF-1), each $\varepsilon^{\text{native}}$ was set to the average of the values applied to the N and N' folding frames. In the context of our simulation model, the WT E65Q and E65'Q constructs are identical in the absence of Ca^{2+}, as the E65Q mutation has a minimal effect on the stability of apo calbindin.[4] Phe→Ala underpacking mutations were modeled by setting $\varepsilon^{\text{native}}$ to a very low value (0.05 kcal mol^{-1}) for native contacts involving the mutated residue.

Parameterization of the protein model To parameterize the protein model, we tuned the values of the well-depth ε to reproduce the experimental folding free energies ΔG^{fold} values of both apo and holo WT calbindin at 20 ^{o}C and pH 7.5; the same ε values were then used for the corresponding CP versions of calbindin. Thus, Ca^{2+}-binding was implicitly modeled by uniformly stabilizing the protein relative to its apo form by an appropriate amount at the experimental [Ca^{2+}] of 100 μM.[4] To maintain a balance between nonlocal and local interactions, the potential barriers V_1 and V_3 for all pseudo-dihedrals not in the linker regions were also scaled by the same factor starting from ε, V_1, and V_3 values that have reproduced the experimental folding free energy of barnase (0.57, 0.475, and 0.2375 kcal mol^{-1}, respectively) [13]. For both apo and holo WT calbindin, the scaling factor was iteratively tuned until steady-state WE simulations yielded a ratio of folding and unfolding rate constants that reproduced the corresponding experimental ΔG^{fold} values ($\Delta G^{\text{fold}} = -RT\ln[k_{\text{fold}}/k_{\text{unfold}}]$). Optimal ε, V_1, and V_3 values are provided in Supplementary Table 1.

To generate pre-equilibrated initial conformations for steady-state WE simulations of the folding and unfolding processes of WT and CP calbindin, equilibrium WE simulations were initialized from the corresponding folded and extended conformations, respectively. The progress coordinate for these simulations was the C_α RMSD of the WT residues relative to the crystal structure of WT calbindin, excluding the six-residue linker unique to CP calbindin. For extensive conformational sampling, the progress coordinate ranged from 0 to 30 Å with a 1-Å bin spacing and a target number of 2 trajectories/bin. Each equilibrium WE simulation was carried out until the probability distribution as a function of the progress coordinate converged, *i.e.* exhibiting no further changes in the positions of the maxima corresponding to the folded and unfolded states. To achieve convergence, simulations starting from the folded and extended conformations were carried out for 500 and 1500 WE iterations (fixed intervals τ), respectively, which correspond to molecular times of 50 ns and 150 ns (Nτ where N is the number of WE iterations). From the set of conformations generated by equilibrium WE simulations, all conformations with

a progress coordinate within 0.1 Å of the position of the probability maximum of a given state were selected for use as initial states in subsequent steady-state WE simulations. In addition, the boundary of each state was defined as the observed position of the probability maximum, based on an estimate of the probability distribution as a function of the progress coordinate. Due to the stochastic nature of equilibrium WE simulations used to generate initial states, the number of initial states varied, exceeding 150 conformations in simulations starting from the folded state and exceeding 750 conformations in simulations starting from the unfolded state.

For steady-state WE simulations of the unfolding and folding processes of WT and CP calbindin, the progress coordinate was the C_α RMSD from the crystal structure of WT calbindin, excluding the six-residue linker region of CP calbindin, and bins were spaced every 0.2 Å between the boundaries of the folded and unfolded states, as defined by equilibrium WE simulations (*e.g.*, 2.2 Å and 12.6 Å, respectively, for WT apo calbindin). In simulations of folding, trajectories were recycled upon reaching the folded state, and in simulations of unfolding, trajectories were recycled upon reaching the target unfolded state. Simulations were carried out for 2000 WE iterations (200 ns molecular time) to achieve a converged estimate of the folding free energy (Supplementary Fig. 2).

WE simulations of switch constructs To generate pre-equilibrated initial states for each switch construct, an equilibrium WE simulation was initialized from the N folded state (and N' unfolded state), and another equilibrium WE simulation was initialized from the N' folded state (and N unfolded state). The initial conformations consisted of a folded frame based on the crystal structure of WT calbindin and an orphan EF-hand in an extended conformation. For simulations of the N folded state, the progress coordinate was the C_α RMSD of the N frame relative to the crystal structure of WT calbindin; for simulations of the N' folded state, the progress coordinate was the C_α RMSD of the N' frame (not including the six-residue linker) relative to the crystal structure of WT calbindin. The progress coordinate ranged from 0 to 30 Å with a 1-Å bin spacing and a target number of 5 trajectories/bin. Each simulation was carried out for 1000 WE iterations (100 ns molecular time) to achieve convergence of the probability distribution as a function of the progress coordinate, *i.e.* positions of the maxima corresponding to the N or N' folded states. As was performed for WT and CP calbindin, all conformations within 0.1 Å of the probability maxima were selected for use as initial states in subsequent steady-state WE simulations of switch constructs. The boundaries of the N and N' folded states were defined as the positions of the probably maxima, where the probability distribution is viewed as a function of the C_α RMSD of the N frame or the C_α RMSD of the N' frame, respectively.

Separate steady-state WE simulations were performed to extensively sample the N → N' and N' → N transitions. Initial states, which were generated by equilibrium WE simulation, comprised an ensemble of N or N' conformations; for all simulations, the number of initial states exceeded 500. A two-dimensional progress coordinate was used consisting of the C_α RMSD of the N frame and the C_α RMSD of the N' frame relative to the crystal structure of WT calbindin, excluding the six-residue linker within the N' frame. To focus sampling on the switching process, the progress coordinate was binned radially, at 1° increments, with edges emanating from the intersection of the lines representing the N and N' probability maxima, as determined by equilibrium WE simulations of the same construct (Supplementary Fig. 5). A target number of 5 trajectories/bin was used, with the additional criterion that split/merge events be performed to maintain a constant number of 576 trajectories to make use of all available computing cores. In simulations of the N → N' switching process, trajectories were recycled upon reaching the target N' folded state, while in simulations of the N' → N switching process, trajectories were recycled upon reaching the target N folded state, as defined by previous equilibrium WE simulations. Simulations were carried out for a total of 2000

WE iterations (200 ns molecular time) to achieve < 50% relative errors in the rate constants, where the errors represent one standard error of the mean (Supplementary Fig. 8).

Propagation of dynamics Dynamics of the WE simulations were propagated at 20 °C using a Brownian dynamics algorithm[14] with hydrodynamic interactions,[15,16] as implemented in the UIOWA-BD software.[13,17] Inclusion of hydrodynamic interactions introduces correlations in the protein dynamics that would have resulted from collisions with solvent molecules and is essential for yielding realistic diffusive properties in the absence of explicit solvent.[17] Hydrodynamic radii were set to 5.3 Å, which has been found to reproduce translational and rotational diffusion coefficients of all-atom models when using the C_α-models of this study.[17] To enable a 50 fs time step, all pseudo-bonds were constrained to native bond lengths using the LINCS algorithm.[18] Pairwise nonbonded interactions were calculated for residues <25 Å apart, updating short-range interactions (< 12.5 Å) every 50 fs, and longer-range interactions (> 12.5 Å) every 500 fs.

Calculation of free energy surfaces. To calculate free energy surfaces of folding/switching processes, we first generated an equilibrium ensemble of trajectories by combining steady-state WE trajectories of the processes in opposite directions and then applied a non-Markovian reweighting procedure,[19] constructing a non-Markovian transition matrix from observed bin-to-bin transitions that accounts for the identity of the state last visited by each trajectory. This history-labeled matrix was solved to yield bin populations for each steady state. The sum of the two steady-state bin populations gives the equilibrium bin populations thereby yielding equilibrium probability densities, and ultimately, the free energy surfaces. The reweighting procedure can be carried out using arbitrary bins and state definitions. Here, we used a rectilinear set of bins along the progress coordinate(s), with mesh sizes of 0.2 Å and 1 Å for simulations of isolated calbindin and switch constructs, respectively. Each free energy surface was generated from 10 steady-state WE simulations in both forward and reverse directions.

Calculation of contact scores The contact score $C_X(i)$ for each residue index i was calculated as

$$C_X(i) = \sum_j p(i,j|X) \tag{4}$$

where the sum is over those residues j that form native contacts with residue i, $p(i,j|X)$ is the conditional probability that residues i and j are in contact given that the protein is in ensemble X, and X is either N, N', the N→N' TPE, or the N'→N TPE. Thus, $C_X(i)$ accounts for the number of residues with which residue i forms contacts as well as the frequency of forming these contacts. To calculate contact scores for the TPE, statistical weights were assigned to each transition path based on its weight entering the target state. Given that a conformation may belong to multiple transition paths (as a result of replication events), the weight for each conformation was defined as the sum of the weights of its successful child trajectories.

Calculation of simulation efficiency As done previously,[20] the efficiency S_k of WE simulations relative to brute-force (BF) simulations in calculating the rate constant k was estimated as:

$$S_k = \frac{t_{\mathrm{BF}}}{t_{\mathrm{WE}}}\left(\frac{\Delta k_{\mathrm{BF}}}{\Delta k_{\mathrm{WE}}}\right)^2 \tag{5}$$

where Δk_{WE} is one standard error of the mean of the rate constant k obtained using WE simulations with a total computing time of t_{WE}, and Δk_{BF} is one standard error of the mean of the rate constant k predicted for brute-force simulations with a total computing time of t_{BF}.

Since it was not feasible to directly compute Δk_{BF} by carrying out BF simulations for sufficiently long times, Δk_{BF} was determined analytically by assuming that the switching process may be modeled as a Poisson process with rate parameter k, as obtained via WE simulations. A derivation of the equation for calculating Δk_{BF} is as follows. Consider a set of n standard simulations of (arbitrary) length t such that the total time of the BF simulation is $t_{\mathrm{BF}} = nt$. Simulations are initiated from state A to calculate the rate constant for the process $A \rightarrow B$. To constrain the simulation to sampling only the process of interest, a simulation is restarted from the initial state A immediately upon reaching the final state B. For simulation times t that are much longer than the maximum duration time of an event, the $A \rightarrow B$ transition is well approximated as a Poisson process on $\mathbb{R}^+$ with rate parameter k. The number of events C_i in the ith simulation is distributed as $C_i \sim Poi(kt)$. To estimate the rate constant k, we evaluate the estimator

$$\hat{k}(\{C_i\}_{i=1}^{n}) \coloneqq \frac{\sum_{i=1}^{n} C_i}{nt}. \tag{6}$$

Note that $\hat{k}$ is unbiased, as

$$\mathbb{E}\left(\hat{k}(\{C_i\}_{i=1}^{n})\right) = \mathbb{E}\left(\frac{\sum_{i=1}^{n} C_i}{nt}\right) = \frac{\sum_{i=1}^{n} \mathbb{E}(C_i)}{nt} = \frac{\sum_{i=1}^{n} kt}{nt} = k, \tag{7}$$

where $\mathbb{E}$ denotes the expected value operator. Finally, Δk_{BF} is given by the square root of the variance of the sample statistic $\hat{k}$:

$$\Delta k_{\mathrm{BF}} = \sqrt{Var\left(\frac{\sum_{i=1}^{n} C_i}{nt}\right)} = \sqrt{\frac{1}{n^2 t^2} Var\left(\sum_{i=1}^{n} C_i\right)} = \sqrt{\frac{n}{n^2 t^2} Var(C_i)} = \sqrt{\frac{nkt}{n^2 t^2}} = \sqrt{\frac{k}{nt}}. \tag{8}$$

The third equality is given by independence of $\{C_i\}_{i=1}^{n}$, which implies also that $\{C_i\}_{i=1}^{n}$ are uncorrelated. Note that in this model, the choice of n and t are inconsequential in the efficiency metric given by Huber and Kim [20]

$$S_k = \frac{t_{\mathrm{BF}}}{t_{\mathrm{WE}}} \frac{\Delta k_{\mathrm{BF}}^2}{\Delta k_{\mathrm{WE}}^2}, \tag{9}$$

as n and t cancel in the numerator:

$$t_{\mathrm{BF}} \Delta k_{\mathrm{BF}}^2 = nt \frac{k}{nt} = k. \tag{10}$$

Thus, while the model only holds for sufficiently long t, S_k does not depend upon nt and so we choose $t_{\mathrm{BF}} = t_{\mathrm{WE}}$ for simplicity of presentation.

Calculation of the number of independent switching events. Events are considered independent if the corresponding trajectories do not share a common simulation segment within the period between the event and one correlation time before the event. The correlation time was determined by monitoring the autocorrelation of the weight flux into the target state as function of the lag time, and identifying the first

lag time that results in zero autocorrelation. In the present study, we calculated the correlation time from the autocorrelation function of the *number* flux, which is the number of successful events per iteration. Thus, trajectories were weighted equally when determining the number flux, which is in contrast to a weight flux, or probability of successful events. The number flux was chosen over the weight flux in order to avoid skewing the correlation time towards that of the higher weight trajectories.

Gene construction. Mutations were introduced to the bovine calbindin D_{9k} genes using the In-Fusion Cloning Plus Kit (Takara Bio). The CP calbindin gene was constructed by ligating the first half of the gene (encoding amino acids 44 to 75, with an additional Cys codon added at the 5' position) and the second half of the gene (encoding amino acids 1 to 43, with an additional 5' sequence encoding a GGAAAM peptide linker). The AFF genes were then assembled by replacing the second half of CP gene with the full-length WT calbindin gene. All genes were fully sequenced.

Protein expression and purification. Proteins were expressed in *E. coli* BL21(DE3) at 20 °C for 16 h after induction with 0.3 mM isopropyl β-D-1-thiogalactopyranoside. Cells were lysed with lysozyme in 10 mM Tris (pH 7.5), 0.1 M NaCl, 1 mM EDTA, 10 mM β-mercaptoethanol (BME). After treating with 5 mM $MgSO_4$ and DNase I to reduce viscosity, 1/10 volume of 1M sodium acetate (pH 4.3) was added and the precipitate was removed by centrifugation. The supernatant was loaded onto a DE52 column (Whatman) equilibrated with 0.1 M sodium acetate (pH 4.8), 0.1 M NaCl, 1 mM EDTA. The flow-through was collected and dialyzed against 10 mM imidazole (pH 6), 1 mM EDTA, 10 mM BME. The solution was loaded onto a Q-Sepharose column (GE Healthcare) equilibrated in the same buffer, and eluted with 0 to 1.0 M NaCl gradient. N and CP forms of calbindin were further purified on a Superdex S75 column (GE Healthcare).

The full sequence of the WT E65Q construct, numbered relative to WT calbindin, is as follows:

M C $^{44'}$S T L D E $^{49'}$L F E E L $^{54'}$D K N G D $^{59'}$G E V S F $^{64'}$E E F Q V $^{69'}$L V K K I S $^{75'}$Q G G A A A M ^{1}K S P E ^{5}E L K G I^{10} F E K Y A^{15} A K E G D ^{20}P N Q L S ^{25}K E E L K^{30} L L L Q T ^{35}E F P S L ^{40}L K G M C S ^{45}T L D E L ^{50}F E E L D ^{55}K N G D G ^{60}E V S F E ^{65}Q F Q V L ^{70}V K K I S ^{75}Q

HSQC NMR spectra. NMR samples were prepared by dissolving lyophilized, uniform ^{15}N-labeled proteins (99 atom %) in 20 mM Tris (pH 7.2), 0.15 M NaCl, 5 mM $CaCl_2$, 5 % $^{2}H_2O$. HSQC spectra were acquired on an 800 MHz Bruker AVANCE III HD NMR spectrometer. Sample concentration was 0.7 mM and temperature was 25 °C. Data were processed using the Bruker TopSpin 4.0.2 analysis package. We gratefully acknowledge David Kiemle for setting up the NMR experiments.

Supplementary References

1. Oktaviani, N. A. *et al.* 100% complete assignment of non-labile 1H, 13C, and 15N signals for calcium-loaded calbindin D_{9k} P43G. *Biomol. NMR Assign.* **5**, 79-84 (2011).
2. Linse, S., Thulin, E. & Sellers, P. Disulfide bonds in homo- and heterdimers of EF-hand subdomains of calbindin D_{9k}: stability, calcium binding, and NMR studies. *Protein Sci.* **2**, 985-1000 (1993).
3. Julenius, K. *et al.* Coupling of ligand binding and dimerization of helix-loop-helix peptides and sedimentation analyses of calbindin D_{9k} EF-hands. *Proteins* **47**, 323-333 (2002).
4. Stratton, M. M., Mitrea, D. M. & Loh, S. N. A Ca^{2+}-sensing molecular switch based on alternate frame protein folding. *ACS Chem. Biol.* **3**, 723-732 (2008).
5. Best, R. B. & Hummer, G. Microscopic interpretation of folding phi-values using the transition path ensemble. *Proc. Natl. Acad. Sci. USA* **113**, 3263-3268 (2016).
6. Sali, A. & Blundell, T. L. Comparative protein modelling by satisfaction of spatial restraints. *J. Mol. Biol.* **234**, 779-815 (1993).
7. Szebenyi, D. M. E. & Moffat, K. The refined structure of vitamin D-dependent calcium-binding protein from bovine intestine. *J. Biol. Chem.* **261**, 8761-8777 (1986).
8. Soto, C. S., Fasnacht, M., Zhu, J., Forrest, L. & Honig, B. Loop modeling: Sampling, filtering, and scoring. *Proteins: Struct., Funct., Bioinf.* **70**, 834-843 (2008).
9. Go, N. Theoretical studies of protein folding. *Annu. Rev. Biophys. Bioeng.* **12**, 183-210 (1983).
10. Takada, S. Gō-ing for the prediction of protein folding mechanisms. *Proc. Natl. Acad. Sci. USA* **96**, 11698-11700 (1999).
11. Clementi, C., Nymeyer, H. & Onuchic, J. N. Topological and energetic factors: What determines the structural details of the transition state ensemble and "en-route" intermediates for protein folding? An investigation for small globular proteins. *J. Mol. Biol.* **298**, 937-953 (2000).
12. Koga, N. & Takada, S. Roles of native topology and chain-length scaling in protein folding: A simulation study with a Gō-like model. *J. Mol. Biol.* **313**, 171-180 (2001).
13. Elcock, A. H. Molecular simulations of cotranslational protein folding: Fragment stabilities, folding cooperativity, and trapping in the ribosome. *PLoS Comput. Biol.* **2**, e98 (2006).
14. Ermak, D. L. & McCammon, J. A. Brownian dynamics with hydrodynamic interactions. *J. Chem. Phys.* **69**, 1352-1360 (1978).
15. Yamakawa, H. Transport properties of polymer chains in dilute solution - hydrodynamic interaction. *J. Chem. Phys.* **53**, 436-443 (1970).
16. Rotne, J. & Prager, S. Variational treatment of hydrodynamic interaction in polymers. *J. Chem. Phys.* **50**, 4831-4837 (1969).
17. Frembgen-Kesner, T. & Elcock, A. H. Striking effects of hydrodynamic interactions on the simulated diffusion and folding of proteins. *J. Chem. Theory Comput.* **5**, 242-256 (2009).
18. Hess, B., Bekker, H., Berendsen, H. & Fraaije, J. LINCS: A linear constraint solver for molecular simulations. *J. Comput. Chem.* **18**, 1463-1472 (1997).
19. Suarez, E. *et al.* Simultaneous computation of dynamical and equilibrium information using a weighted ensemble of trajectories. *J. Chem. Theory Comput.* **10**, 2658-2667 (2014).
20. Huber, G. A. & Kim, S. Weighted-Ensemble Brownian dynamics simulations of protein association reactions. *Biophys. J.* **70**, 97-110 (1996).
