# Supplementary material for: Large enhancement of response times of a protein conformational switch by computational design
Source: Nat Commun. 2018 Mar 9;9:1013. doi: 10.1038/s41467-018-03228-6 (PMC5844902; doi:10.1038/s41467-018-03228-6)
Supplement: Supplementary file 3 — Description of Additional Supplementary Files [file 41467_2018_3228_MOESM3_ESM.pdf]

Description of Supplementary Files

File Name: Supplementary Movie 1
Description: **A representative pathway of the N → N′ switching process for the E65Q construct, generated by WE simulations.** The pathway corresponds to the blue line in Fig. 2a. The red, green, and blue regions correspond to the EF-2′, EF-1, and EF-2 hands, respectively, and the gray region represents the six-amino acid linker between the EF-2′ and EF1 hands in the CP/N′ frame that connects the original N- and C-termini of the original WT/N frame. For reference, the yellow spheres indicate the positions of the BODIPY fluorophores, which were used in the experiments but not included in the simulations. The probability p (statistical weight) of each snapshot along the pathway is indicated in the lower right hand corner. The E65Q construct begins in the N folded state, in which the green and blue regions are folded together. The red region (EF-2′) displaces the blue region (EF-2) as the switch transitions to the N′ folded state.

File Name: Supplementary Movie 2
Description: **A representative pathway of the N′ → N switching process for the E65′Q construct, generated by WE simulations.** The pathway corresponds to the blue line in Fig. 2c, and the color scheme and nomenclature are the same as Supplementary Movie 1. The E65′Q construct begins in the N′ folded state, in which the red and the green regions are folded together. The blue region (EF-2) displaces the red region (EF-2′) as the switch transitions to the N folded state.
